# Late-onset efavirenz toxicity: A descriptive study from Pretoria, South Africa

**DOI:** 10.4102/sajhivmed.v24i1.1439

**Published:** 2023-01-12

**Authors:** Lyneshree Munsami, Clara M. Schutte, Maryke de Villiers, Juliane Hiesgen

**Affiliations:** 1 Department of Neurology, Faculty of Health Science, University of Pretoria, Pretoria, South Africa; 2 Department of Internal Medicine, Faculty of Health Science, University of Pretoria, Pretoria, South Africa

**Keywords:** HIV, efavirenz toxicity, ataxia, encephalopathy, psychosis

## Abstract

**Background:**

The neuropsychiatric side effects of efavirenz occur mainly early during treatment and are usually mild. A lesser-known and serious complication is late-onset efavirenz toxicity causing ataxia and encephalopathy. Data regarding this condition are limited.

**Objectives:**

We describe the clinical picture of late-onset efavirenz toxicity, investigate co-morbidities and report outcomes.

**Method:**

This descriptive study of all patients with late-onset efavirenz toxicity was conducted over three years at Kalafong Provincial Tertiary Hospital, Pretoria, South Africa.

**Results:**

Forty consecutive patients were identified. Mean age was 42.1 years, three patients (7.5%) were male and the mean efavirenz level was 49.0 μg/mL (standard deviation [s.d.]: 24.8). Cerebellar ataxia (82.5%) and encephalopathy (47.5%) were the most common presenting features (40.0% had both); four patients presented with psychosis. Presence of encephalopathy and/or cerebellar ataxia was associated with higher efavirenz levels compared with psychosis (52.1 μg/mL, s.d.: 24.1 vs 25.0 μg/mL, s.d.: 17.1). In most patients, symptoms resolved, but four patients (10.0%) died, and one patient remained ataxic.

**Conclusion:**

Late-onset efavirenz toxicity typically presented with ataxia and encephalopathy, but psychosis can be the presenting feature. The outcome after withdrawal was good, but the mortality of 10.0% is concerning. Recent changes in guidelines favour dolutegravir, but many patients remain on efavirenz, and awareness of the condition is vital.

**What this study adds:**

This large, single-centre study contributes to the limited data of HIV-positive patients with late-onset efavirenz toxicity and emphasises its ongoing relevance in clinical practice.

## Introduction

With more than 8.2 million people living with HIV/AIDS in 2021, South Africa contributes approximately 21% to the global burden of the disease.^1^ About 71% of the HIV-infected population in South Africa receive antiretroviral treatment (ART).^2^ Over the years, different ART regimens have been used because serious side effects of early antiretroviral drugs had caused significant morbidity and mortality and better drugs became available.^3^ The non-nucleoside reverse transcriptase inhibitor (NNRTI) efavirenz was, until recently, the preferred component of first-line ART. It is metabolised predominantly by the cytochrome P450 enzyme *CYP2B6*. Loss of function polymorphisms in the *CYP2B6* gene increase the plasma concentrations of efavirenz^4^ and play a role in the interaction of efavirenz with other antiretroviral drugs or anti-tuberculous (TB) medication.^5^

Efavirenz commonly causes early neuropsychiatric toxicity, which is usually not severe enough to warrant discontinuation of the drug and typically resolves despite continuing efavirenz.^6,7^ However, from around 2016, symptoms of severe efavirenz toxicity with a delayed onset were recognised in adults and children.^8,9,10^ Cerebellar ataxia and encephalopathy constitute the main features of this new clinical syndrome, which was coined ‘late-onset’ toxicity.^11^ It was soon evident that these findings occurred especially in women and children associated with a low body weight. Polymorphisms in the *CYP2B6*, have also been associated with slower efavirenz metabolism and toxicity.^12^ If not recognised early, these side effects can lead to significant morbidity and to the death of patients.^8^

In 2017, the South African guidelines for first-line ART were updated.^13^ Efavirenz has been replaced by dolutegravir since 2020. Efavirenz is still recommended for patients with tuberculosis and many patients have opted to stay on efavirenz if they are doing well. A recent study reported a slightly higher prevalence of neural-tube defects and major external structural defects in babies born to mothers exposed to dolutegravir compared to other types of ART, but this could not be confirmed in other studies.^14,15,16^ A South African modelling study found an overall benefit of dolutegravir compared to efavirenz, due to fewer deaths among women and less overall HIV transmission, despite a higher risk of neural-tube defects.^17^ Thus, considering benefits, risks and costs, dolutegravir-based regimens are now preferred for ART initiation, including in women intending pregnancy, in sub-Saharan Africa.^18,19^ Nevertheless, some women of childbearing potential might, after adequate counselling about the individual risks and benefits, opt against dolutegravir during pregnancy. Efavirenz remains a good first-line ART option for individuals who do not tolerate dolutegravir, or where this drug is contraindicated or declined by the patient.^19^

At Kalafong Provincial Tertiary Hospital (KPTH), we have seen an increasing number of patients with ataxia and encephalopathy due to late-onset efavirenz toxicity and still encounter them despite the widely implemented new ART regimen. With this study, we aim to characterise the clinical spectrum of late-onset efavirenz toxicity.

## Objectives

The objectives of this study were to identify patients with late-onset efavirenz toxicity seen at KPTH and describe their clinical presentation. In addition, we aimed to collect data about co-morbidities and outcomes and compare these findings with results from other studies on this topic. Finally, efavirenz levels were compared between different subgroups in our case series.

## Methods

This descriptive cohort study was conducted at KPTH, a tertiary academic hospital affiliated to the University of Pretoria, South Africa. Data were collected over a three-year period, with a retrospective (01 January 2018 – 31 December 2019) and a prospective arm (01 January 2020 – 31 December 2020).

We included all adult inpatients and outpatients with HIV on an efavirenz-based ART regimen with neuropsychiatric symptoms and signs consistent with late-onset efavirenz toxicity and laboratory-confirmed toxic efavirenz levels. For the retrospective period, discharge summaries from patients admitted to the hospital were screened, files were retrieved from the records department and data transferred into a standardised and anonymised data collection sheet. Additionally, a list of all patients with toxic efavirenz levels done at our institution was obtained from the National Health Laboratory Service (NHLS) and their files were scrutinised, and if the patients had symptoms of efavirenz toxicity, they were included and their data extracted. We considered neuropsychiatric symptoms to be associated with toxic efavirenz levels if no other cause was identified. Routine investigations for encephalopathy were undertaken. In the prospective arm, we obtained written consent for study participation before data collection. All participants in the prospective arm and most in the retrospective arm were assessed by a neurologist. We excluded individuals with toxic efavirenz levels due to intentional overdose or without neuropsychiatric symptoms of toxicity.

Efavirenz levels were quantified at NHLS at Charlotte Maxeke Johannesburg Academic Hospital using ultra-high performance liquid chromatography with tandem mass spectrometry.

Data collected for this study included demographic data, laboratory and imaging results, data about clinical presentation, course of disease and outcome. Data were exported to a Microsoft Excel sheet and results analysed using statistical software package IBM^®^ Statistical Package for Social Sciences (SPSS) version 28. We used frequencies for descriptive statistics; for efavirenz levels, the normality of the distribution was tested with Shapiro-Wilks test and Q-Q plot. For normally distributed data, variables were reported as means and standard deviation.

### Ethical considerations

Data collection started after ethical clearance was granted from the University of Pretoria (Research Ethics Committee of the Faculty of Health Sciences) and from the Research Committee of KPTH (reference number: 224/2020). Participation was voluntary and all participants in the prospective arm provided written informed consent. In individuals with encephalopathy or psychosis, the consent was obtained when the person had regained the ability to consent. In addition, a special form for ‘consent by proxy’ was approved by the Ethics Committee. The data were anonymised to ensure the privacy and confidentiality of the participants’ information.

## Results

### Demographics

Over the three-year period, we identified 47 participants with efavirenz levels in the toxic range (> 4 μg/mL). Seven patients were excluded: one had intentionally overdosed on his HIV medication; four had no neuropsychiatric symptoms, and two only had a polyneuropathy with no central nervous system symptoms. (A flow chart illustrating the exclusion process is available as Online Appendix 1). Twenty-nine participants were recruited retrospectively (2018–2019) and the remaining 11 prospectively in 2020.

Most of the participants were female (*n* = 37, 92.5%). The mean age was 42.1 years (standard deviation [s.d.]: 12.6), with three participants younger than 25 (7.5%) and one older than 65 (2.5%).

### HIV-related data

The demographic characteristics and HIV baseline data are summarised in Table 1. Regarding the duration of ART use, 17 individuals were on an efavirenz-based regimen for one year or less, representing 42.5% of the participants with a known duration of HIV treatment. The others were on treatment for more than one year. Most of the participants (*n* = 26, 65.0%) had CD4 counts of greater than 200 cells/μL, with nine (22.5%) showing a normal CD4 count above 500 cells/μL. The HIV viral load was suppressed in 85.0% of participants.

**TABLE 1:** Demographic characteristics and HIV-related data of patients with late-onset efavirenz toxicity.

Variable	*n* (40)	Mean	s.d.
**Gender**			
Female	37	-	-
Male	3	-	-
Age in years	-	42.1	12.6
Female	-	42.2	12.8
Male	-	41.3	13.1
**Duration of HIV infection**			
≤ 1 year	12	-	-
> 1 years to ≤ 2 years	1	-	-
> 2 years to ≤ 5 years	11	-	-
> 5 years to ≤ 10 years	6	-	-
> 10 years	5	-	-
Unknown duration	5	-	-
**Duration of antiretroviral treatment**			
≤ 1 year	17	-	-
> 1 year to ≤ 2 years	1	-	-
> 2 year to ≤ 5 years	9	-	-
> 5 years to ≤ 10 years	5	-	-
> 10 years	2	-	-
Unknown duration	6	-	-
**CD4 cell count (cells/μL)**			
< 100	4	-	-
100–199	9	-	-
200–499	17	-	-
> 500	9	-	-
Unknown	1	-	-
**HIV viral load (copies/mL)**			
Suppressed (< 20)	34	-	-
20–999	0	-	-
> 1000	4	-	-
Unknown	2	-	-

s.d., standard deviation.

### Clinical presentation

The majority of participants (*n* = 36) presented with cerebellar ataxia and/or encephalopathy. Out of 33 participants who presented with cerebellar signs, 17 did not have encephalopathy; they were ataxic but fully alert, without cognitive impairment. Most of the 19 participants with encephalopathy also had cerebellar signs, with only three patients being encephalopathic without ataxia.

Four participants (10%) presented with psychosis without encephalopathy and only one of those had ataxia. Only two participants in the retrospective arm had nystagmus as part of the cerebellar presentation. No participant in the prospective arm had nystagmus. Four individuals (10%) had additional features of distal symmetrical polyneuropathy at presentation. All of these had identifiable causes (vitamin B12 deficiency, isoniazid [INH] treatment) for a neuropathy in addition to the underlying HIV infection itself. Two patients had additional neurological symptoms; one had tetraparesis (with encephalopathy) and one hemiparesis (with ataxia). Table 2 illustrates the clinical presentation of the participants.

**TABLE 2:** Clinical presentation of patients with late-onset efavirenz toxicity.

Clinical signs	*n*
Ataxia (or other cerebellar signs)	33
Encephalopathy	19
Ataxia and encephalopathy	16
Psychosis	4
Polyneuropathy	4
Others†	2

Note: Some patients with more than one sign.

† , Other presentations included one participant with hemiparesis and one with tetraparesis.

### Efavirenz levels

The mean efavirenz level for all participants was 49.0 μg/mL (s.d.: 24.8) with a minimum level of 8.0 μg/mL, and a maximum level of 96.0 μg/mL. This level is about 12 times higher than the upper limit of the therapeutic range of efavirenz, defined as 1.0 μg/mL – 4.0 μg/mL. The mean efavirenz levels of different subgroups are shown in Table 3.

**TABLE 3:** Efavirenz levels in different subgroups.

Variable	Mean (μg/mL)	*n*	s.d.
Total group	49.0	40	24.8
Ataxia (only)	55.3	33	22.4
Encephalopathy (only)	51.5	19	25.7
Encephalopathy/ataxia	52.1	36	24.1
Psychosis	25.0	4	11–47
Tuberculous (TB) treatment/Isoniazid (INH)	58.1	18	23.5
INH (only)	64.0	9	11.7

s.d., standard deviation.

### Co-infection and co-medication

The most common co-infection was TB, which was found in nine participants. One participant had serological evidence of a cryptococcal infection, but the cerebrospinal fluid (CSF) was negative for all cryptococcal tests, and two patients had syphilis. Table 4 summarises co-infections and co-medications.

**TABLE 4:** Co-infections and co-medication at presentation in patients with late-onset efavirenz toxicity.

Variable	*n*
**Infection**	
Tuberculosis	9
Syphilis	2
*Cryptococcus neoformans*	1
Cytomegalovirus	1
**Medication**	
Anti-tuberculous medication†	9
Isoniazid prophylaxis‡	9

† , Anti-tuberculosis treatment consisted of isoniazid, rifampicin, ethambutol and pyrazinamide in first 2 months and isoniazid and rifampicin from the third month;

‡ , isoniazid 300 mg daily.

### Additional investigations

Thirty-three patients underwent cerebral imaging with computerised tomography (CT) and out of those, 11 later had magnetic resonance imaging (MRI) to exclude an alternative diagnosis. Twenty-four of the CT scans were normal. Nine CT scans showed abnormalities, three in keeping with general cerebral atrophy, three scans had small granulomata or calcifications, and two showed small hypodensities suggestive of old infarcts. One scan revealed a small, supratentorial space-occupying lesion, which we assessed to be most likely a tuberculoma (the patient had TB, including TB meningitis). Out of 11 MRI scans, only one showed an additional finding, namely that of cerebellar atrophy in the participant with persistent ataxia.

Thirty-four participants had a lumbar puncture (LP) performed and out of these, the CSF was normal in 28. The CSF was abnormal in six cases (three showed non-specific abnormalities probably due to the underlying HIV infection: one had mild pleocytosis and two increased protein levels; furthermore, one had a positive treponema pallidum haemagglutination test and two a combination of pleocytosis and elevated protein, of which one had TB meningitis). Nine participants had an electroencephalogram (EEG) recorded, all of them were pathological. Eight recorded diffuse slowing in keeping with encephalopathy, and one showed an epileptiform dysfunction.

Blood results showed abnormalities in 16 participants (40%) and mainly consisted of abnormal liver function tests (LFT). Out of 12 individuals with abnormal LFTs, only one had severe liver impairment. Other abnormalities included two cases with positive syphilis serology, one with abnormal thyroid stimulating hormone (TSH) levels, and one with a positive serum cryptococcal antigen test.

### Outcome

Efavirenz was provisionally stopped in all participants with suspected toxicity and later discontinued, once the supratherapeutic levels were confirmed. In participants with suppressed HIV viral loads, a single drug switch was done from efavirenz to either lopinavir/ritonavir (LPV/r) or atazanavir/ritonavir (ATV/r), depending on the lipogram. In those instances where the viral load was not suppressed, a regimen change to zidovudine/lamivudine (AZT/3TC) and either LPV/r or ATV/r was performed.

Of the 36 individuals discharged from KPTH, 32 were asymptomatic at discharge. Participants on average improved after two weeks without efavirenz. Only one participant recovered in less than one week; three participants needed 3–4 weeks to improve. The remaining four patients had mild residual symptoms at discharge and at 6-month follow up. One participant remained severely ataxic, unable to mobilise independently and requiring a wheelchair and full assistance in her activities of daily living. Her MRI of the brain was abnormal, showing cerebellar atrophy and we investigated her extensively for alternative causes of cerebellar disease.

Four patients passed away during the hospital admission. The deaths were most likely related to the efavirenz toxicity because these patients were encephalopathic and ataxic and were bedridden, with complications secondary to these factors leading to their death.

## Discussion

With this study, we contribute to the limited data about late-onset efavirenz toxicity in HIV-positive patients. To the knowledge of the authors, this case series is the largest single-centre study describing clinical presentation and outcome of these patients.

Most side effects of efavirenz manifest soon after initiation of the drug with central nervous symptoms such as dizziness, sleep disturbances, depression and anxiety.^7^ Often, these symptoms are mild and transient, but more severe cases have been reported.^20,21^ In addition, a study using data from four AIDS clinical trial group studies in the United States found a twofold increased risk for suicidality with an efavirenz-containing antiretroviral regimen compared to a regimen without efavirenz.^22^

Only recently, an association between efavirenz toxicity and a delayed onset of ataxia and encephalopathy in adults and children has been reported.^8,9^ In these reports, high levels of efavirenz were detected and an algorithm for the management of late-onset neurotoxicity, including change of the antiretroviral regimen, was proposed.^11^

Although further research regarding the underlying mechanisms involved in neurotoxicity of efavirenz is needed, there is some evidence indicating that disturbances in mitochondrial function and bioenergetics of cells in the central nervous system are important factors.^23^ The major metabolite of efavirenz, 8-hydroxy-efavirenz, was found to be toxic in neuron cultures and in humans. Altered calcium homeostasis, decreased creatine kinase in the brain, mitochondrial damage, as well as increased pro-inflammatory brain cytokines and cannabinoid system involvement may all be potential mechanisms in the development of toxicity.^24^ Other, recent studies assume a more direct effect of efavirenz. In a study using pharmacokinetic simulations, the efavirenz concentrations in brain tissue were found to be higher compared to plasma or CSF concentrations and in a recent study of patients with late-onset efavirenz toxicity, the 8-hydroxy-efavirenz levels were even decreased.^12,25^

In the first case series of patients with late-onset efavirenz toxicity, the authors already observed a female preponderance and a strong association with low body weight.^8^ These risk factors were confirmed by other authors, and later the concomitant use of INH was found to increase efavirenz levels and result in neuropsychiatric toxicity.^11^ A recent study of 15 patients with late-onset efavirenz toxicity found that all had polymorphisms of *CYP2B6* which result in slower metabolism of efavirenz.^12^ Isoniazid inhibits the CYP2A6 enzyme, which is an accessory pathway to metabolise efavirenz. Although usually only a small proportion of efavirenz is metabolised via this enzyme, it becomes more important in slow metabolisers.^26,27^ INH co-medication has been shown to be an important additional risk factor for the development of efavirenz-associated toxicity.^11^

The efavirenz levels in our participants varied from just above therapeutic level to almost 100.0 μg/mL, with a mean efavirenz level of 49.0 μg/mL. Reports from other centres show similar efavirenz levels as found in our study – Variava et al. reported supratherapeutic levels in 19/20 patients (more than twice the upper limit of normal), with 15 showing concentrations that exceeded the upper limit of assay detection which was 20 mg/L in their laboratory.^8^ In the study by Van Rensburg et al., the median efavirenz plasma concentrations were 50.5 (47.0–65.4) μg/mL in participants with late-onset efavirenz toxicity, which was almost identical to our results.^12^

Consistent with other studies, cerebellar ataxia was the most common presenting clinical sign, found in 33 participants (82.5%). Nineteen (47.5%) had some degree of encephalopathy, which varied from confusion to coma. Most of the participants with encephalopathy also had ataxia, with only three patients being encephalopathic without ataxia. Variava et al. reported similar findings, where 11 of 20 patients had encephalopathy with ataxia and the remaining patients had isolated ataxia.^8^

Variava et al. found that no patient in their case series of 20 with severe cerebellar ataxia had nystagmus. Cross et al. found nystagmus in four out of seven patients, while Van Rensburg et al. reported nystagmus in two out of 15 participants with late-onset efavirenz toxicity.^11,12^

In our case series, only two participants out of 33 with cerebellar ataxia were found to have nystagmus.

Four of our participants had acute psychosis as the presenting feature. These individuals did not show a reduced level of consciousness or confusion but had delusions and hallucinations. One out of these four had cerebellar ataxia. Cross et al. described two patients who had a mood disorder and psychotic symptoms prior to their presentation with ataxia and encephalopathy, one requiring admission to a psychiatric ward.^11^ In 2002, Sabato et al. reported a female patient with a previous history of mental illness, who was on antipsychotic therapy and benzodiazepines, developing severe psychosis and catatonia associated with toxic efavirenz levels.^20^ Other isolated case reports with acute psychosis, have been reported.^28,29^ The possibility of psychosis as a clinical manifestation of late-onset efavirenz toxicity should be further investigated, and we suggest that psychosis occurring in patients on efavirenz-containing ART should prompt measuring efavirenz levels.

Although four patients had polyneuropathy as an additional clinical sign, we do not consider this as a clinical presentation directly related to efavirenz. There was no temporal association of the neuropathy and the efavirenz toxicity and alternative causes for a neuropathy were found in all patients, in addition to the underlying HIV infection.

Regarding the outcome, we found similar results as previous reports of patients with late-onset efavirenz toxicity. Most patients, 80%, had a resolution of symptoms after discontinuation of efavirenz while still in the hospital, usually within two weeks. Four patients had residual symptoms at the time of discharge, but only one patient remained severely ataxic, unable to walk unassisted and requiring help in all activities of daily life.

Unfortunately, four participants (10%) passed away during their hospital stay. This corresponds to data from Variava et al.: in their cohort, three out of 20 women died.

This study has some limitations. As a combined retrospective and prospective analysis, complete data were not available for all patients. For example, weight was not measured in the retrospective study period. In addition, we did not test for *CYP2B6* genotypes and, therefore, do not know what proportion of our patients were slow metabolisers. As in previous studies of patients with late-onset efavirenz toxicity, our number of participants is relatively low. Therefore, although we found trends comparing efavirenz levels of different subgroups, further statistical analysis was limited due to the small numbers.

## Conclusion

With this single-centre study, we contribute 40 patients with late-onset efavirenz toxicity, adding to the limited data currently available. Although female gender was predominant, male patients may also present with efavirenz toxicity. Our results validate an association between efavirenz toxicity and INH. In addition to ataxia and encephalopathy, psychosis might be a presenting feature in late-onset toxicity and should prompt efavirenz level testing.
